# Through the Newsfeed Glass: Rethinking Filter Bubbles and Echo Chambers

**DOI:** 10.1007/s13347-021-00494-z

**Published:** 2022-03-15

**Authors:** Giacomo Figà Talamanca, Selene Arfini

**Affiliations:** 1 Department Ethics and Political Philosophy, Radboud University, Nijmegen, The Netherlands; 2 Department of Humanities - Philosophy Section, University of Pavia, Pavia, Italy; 3 Computational Philosophy Laboratory, University of Pavia, Pavia, Italy

**Keywords:** Filter Bubbles, Echo Chambers, Epistemic Bubble, Ignorance Bubble, Epistemic Discomfort, Digital Environments

## Abstract

In this paper, we will re-elaborate the notions of filter bubble and of echo chamber by considering human cognitive systems’ limitations in everyday interactions and how they experience digital technologies. Researchers who applied the concept of filter bubble and echo chambers in empirical investigations see them as forms of algorithmically-caused systems that seclude the users of digital technologies from viewpoints and opinions that oppose theirs. However, a significant majority of empirical research has shown that users do find and interact with opposing views. Furthermore, we argue that the notion of filter bubble overestimates the social impact of digital technologies in explaining social and political developments without considering the not-only-technological circumstances of online behavior and interaction. This provides us with motivation to reconsider this notion’s validity and re-elaborate it in light of existing epistemological theories that deal with the discomfort people experience when dealing with what they do not know. Therefore, we will survey a series of philosophical reflections regarding the epistemic limitations of human cognitive systems. In particular, we will discuss how knowledge and mere belief are phenomenologically indistinguishable and how people’s experience of having their beliefs challenged is cause of epistemic discomfort. We will then go on to argue, in contrast with Pariser’s assumptions, that digital media users might tend to conform to their held viewpoints because of the “immediate” way they experience opposing viewpoints. Since online people experience others and their viewpoints as material features of digital environments, we maintain that this modality of confronting oneself with contrasting opinions prompts users to reinforce their preexisting beliefs and attitudes.

## Introduction

The notion of filter bubble was prominently introduced in digital studies by entrepreneur and Internet activist Eli Pariser (2011). With the term “filter bubble,” Pariser designates a state of intellectual isolation determined by the preference algorithms that underlie contemporary web-based platforms such as Facebook and Google. Online users would be isolated, or “embubbled,” in the sense that they would consume content and interact with communities only *when in accordance* with their previous beliefs. In this way, they would tendentially be excluded from information sources and people in disagreement with their own perspective. They would, in other words, be limited to interact and consume information in an “ideologically safe” and unchallenged environment due to preference algorithms. In a similar vein, Cass Sunstein (2001, 2017; Sunstein and Vermeule, 2009) argues that the advent of web-based platforms favored the creation of intellectually secluded communities deprived of contrary perspectives, or *echo chambers*. For both authors, the diffusion of online platforms and of the algorithms that underlie their functioning lead to a significant reduction in political exchange between citizens of diverging opinions and increasing polarization within them, undermining the foundations of a fair and democratic society. Although the cause–effect relationship between the existence of both filter bubbles and echo chambers and the ruining of democratic discussions online might appear not just convincing, but potentially catastrophic, we aim to question this cause–effect relation that Pariser and Sunstein argue for.

In this paper, indeed, we will discuss and challenge Pariser’s notion of filter bubble and some of Sunstein’s claims on echo chambers, by proposing the re-elaboration of these notions in light of already established theories dealing with some epistemological downsides of fixating beliefs^1^. We argue that a distinction between technological and epistemological embubblements (the latter will be presented in Section 3) is necessary in order to account for a more complex relation between people’s epistemic status and their interaction with digital technologies. We will provide a novel reconceptualization of the concept of filter bubble that can account for such complex relation and that is more fitting with existing empirical research on online information consumption behavior and the intellectual isolation Pariser and Sunstein are concerned with. Our reconceptualization will propose that such intellectual isolation does not derive from the activities of the algorithm alone, but rather from the interaction between the user’s beliefs and cognitive profile and the platform’s interface, which lacks the contextual norms and socio-emotional cues that would make facing contrary viewpoints more functional.

The paper will proceed as follows. In Section 2, we will present the notion of filter bubble in Pariser’s formulation. Specifically, we will discuss its relation to the notion of echo chamber (Sunstein, 2001; Sunstein and Vermeule 2009) and we will argue that both insufficiently provide explanations for the phenomena they aim to account for since there is a lack of empirical evidence that supports them and, they generally favor misunderstandings regarding the impact of technological developments on society. In Section 3, we will lay the grounds for a reformulation of those concepts, by reviewing previous theories of epistemological “embubblement,” elaborated by Woods (2005), Magnani (2011) and Arfini (2019).^2^ For these authors, people are normally subjected to different kinds of embubblement, intrinsic to some of their cognitive processes, such as the “fixation” on certain beliefs, their tendency to follow moral norms, and their underestimating their own ignorance. In Section 4, we will revise the notions of filter bubble and echo chambers as “techno-epistemological” ones, in the light of the previously laid out theories. The definition of such mechanisms will not be a consequence of a reduction of a technological embubblement to an epistemological one (similar to the one already performed by Pariser): we will indeed argue that the filter bubble (as well as online echo chambers) should be understood as the result of people’s “natural” embubblement—understood as a series of default and ultimately inescapable conditions of human agents that we will present in Section 3—while interacting with digital environments. Specifically, Internet users often stumble upon opinions and viewpoints that contradict theirs, and it might strengthen their own beliefs due to what we will define as the “immediacy” of these encounters. This immediacy is the result of a characteristic of many mainstream digital platforms, known as context collapse—which we will also comment on subsection 4.2 (Marwick and boyd, 2011; Vitak, 2012; Costa, 2018).^3^ So, we argue that the unmediated way users see opinions of other people that openly contradict their own views prompt them to a form of cognitive rigidity—i.e., the tendency to fixate on one’s own beliefs and to take them as true until proven contrary^4^—instead of openness and debate. These considerations will shift the focus of “the filter bubble” from technology alone to the interaction between believers and technology, leading to the conclusion that, in a sense, the filter bubble is “already in your head.”

## The Filter Bubble: Relevance and Shortcomings

### The Filter Bubble Thesis

Pariser uses the term filter bubble to describe the impact of personalized search algorithms on Internet platforms such as Google and Facebook. The emergence of these developments, which occurred in the late 2000, marked the advent of what is called “the Web 2.0” (O’Reilly 2007), which enabled Internet-based platforms and services to be more focused on interactivity, social networking and user-generated content. Indeed, Pariser argues that the filter bubble is a fundamental part of Web 2.0 since it results from the personalization process enacted by web-based platforms in order to (allegedly) enhance user engagement, interconnectivity, and content consumption. He argues that “[t]he new generation of Internet filters looks at the things you seem to like … and tries to extrapolate. They [i.e., the algorithms] are prediction engines, constantly creating and refining a theory of who you are and what you’ll do and want next. Together, these engines create a unique universe of information for each of us—what I’ve come to call a filter bubble—which fundamentally alters the way we encounter information online” (Pariser 2011, 9).^5^

A downside of this technological development is, according to Pariser, the intellectual isolation of Internet users: “you’re the only person in your bubble. In an age when shared information is the bedrock of shared experience, the filter bubble is a centrifugal force, pulling us apart” (p. 10).

This condition of intellectual isolation is determined by the following process:

1. Users engage with certain content on the Web, through search engine services like Google or SNSs such as Twitter or Facebook;
2. The algorithms underlying those platforms identify the content preferred by users;
3. Algorithms will provide new contents based on users’ preferences, that is, on their previous engagements with specific matters instead of those they did not engage with.^6^

This mechanism is in line with common understandings of the functioning of intelligent software: the algorithm chooses a set of possible actions to be presented for users, who then choose one of the possible courses of action and the algorithm can further favor engagement by providing other possible actions based on the user’s past choices (Burr et al., 2018). The logic of the filter bubble thesis is furthermore grounded in the service provider’s economic gain. As Srnicek (2017) points out, the aim of platforms such as Facebook, Twitter, or Google—i.e., digital infrastructures that enable agents to interact and act as intermediaries between customers, advertisers, service providers, producers, suppliers and physical objects and events (43)—is the gathering of data regarding their users’ preferences and activities, which is in turn the online service provider’s source of revenue. This is especially so for advertising platforms who sell the extracted data through users’ online activities to advertisers—and it is this kind of platforms that the filter bubble thesis primarily targets, services like Google, Facebook, or Twitter. If these companies aim at data gathering, and if the more data they get the more they can capitalize, the filter bubble thesis seems to fit very well in this dynamic—for, at least at a first glance, the quickest way to get user engagement would be to provide content they (seem to) like or have engaged with before.

Pariser provides an example in order to show the dangers this mechanism can entail. Suppose you have friends on Facebook of different political orientations; and suppose that, as you are more inclined to consume content on the left side of the political spectrum, you visualize and engage with posts by those friends whose political views are closer to yours. What happens, Pariser argues, is that the algorithms underlying Facebook’s newsfeed will take note on the fact that you engage more with content on one side of the political spectrum and less with the other: from there, the algorithm will provide you with content similar to what you previously consumed, by presenting on your newsfeed more posts on the left side of the political spectrum and not from the right.^7^

To summarize, preference algorithms, Pariser argues, accommodate users by providing content consistent with what they consumed in the past: in the example he provides, the user who mainly engages with news consistent with her political views will not receive those from contrary or dissimilar political standpoints. The preference mechanism enacted by the algorithms of contemporary web-based services would lead to an informational embubblement: algorithms would filter information inconsistent with users’ online engagements, so that they would not encounter information opposing their previously shown convictions and beliefs.

It is easy to see the detrimental side of this scenario, *were it true*. If indeed the algorithmic structure of web-based platforms leads to an informational embubblement (personalized for each user’s preferences and engagements) users would not encounter standpoints and information that challenge their standing attitudes and beliefs. If this were to be the case, users of digital platforms would likely become more hardened in their beliefs and political attitudes; this radicalization would, in turn, lead to increasing polarization between different standpoints. The lack of interaction with people of different standpoints would lead to a complete lack of common ground where a minimal degree of mutual understanding would be possible, a necessary feat for a functioning democratic society. Specifically, if a democracy properly functions through the existence of diverse standpoints and the dialogue between them, the creation of ideologically segregated communities would hinder the very capacity for democratic debate. The relevance of this topic is apparent since, beyond Pariser’s account, various authors have tackled and discussed the relationship between platform capitalism’s algorithms and online polarization (Gurumurthy & Bharthur, 2018; Marciano, et. al. 2020; Riemer & Peter, 2021).

As Pariser argues, this mechanism would have deep implications that do not just involve political interaction, but also the state of mind of individual citizens and the general social and political role of these platforms in the years to come. Or as he puts it: “[w]hile the Internet offers access to a dazzling array of sources and options, in the filter bubble we’ll miss many of them. While the Internet can give us new opportunities to grow and experiment with our identities, the economics of personalization push toward a static conception of personhood. While the Internet has the potential to decentralize knowledge and control, in practice it’s concentrating control over what we see and what opportunities we’re offered in the hands of fewer people than ever before” (218). The conclusion, it is argued, is that web-based platforms have severe consequences for democracy and individual and collective well-being, since the preference algorithms that underlie them have the undesirable effect of secluding and polarizing users depending on their attitudes and beliefs.

### Filter Bubbles and Echo Chambers

The notion of filter bubble and Pariser’s thesis regarding its existence and impact is not a solitary instance when it comes to theorizing the (potentially catastrophic) impact of digital technologies in society. The notion of filter bubble is in fact closely associated with what Cass Sunstein (2017) calls “echo chambers.” Sunstein argued that users of the “Web 2.0” would have the “power of personalization, or to create gated communities” (Sunstein, 2017 p. 5) composed by people sharing identical or similar beliefs and convictions. Only previously shared and accepted opinions would be shared in these communities, and beliefs and attitudes challenging those opinions would be excluded. The term “echo chamber” denotes a social network (i.e., a community of people with social ties with one another) who share a (set of) opinion(s) while not interacting with opinions and viewpoints that would contradict them.^8^ Whereas echo chambers can actually exist in offline settings,^9^ on the Internet they would skyrocket in membership numbers and have a much more significant impact than they would in their offline counterparts.

Despite some similarities between filter bubbles and echo chambers, there are two clear differences between the two notions. Firstly, filter bubbles describe forms of intellectual isolation exclusively caused by algorithms (which inferences are based on users’ choices). In contrast, echo chambers are enacted by users themselves. They can exist in offline settings, but (Sunstein argues) are widened in online ones: users of digital technologies would interact only with people who share their same beliefs and values and exclude diverse perspectives (also thanks to algorithmic intercession).

Secondly, the two notions apply at different levels of abstraction: Pariser’s filter bubble aims at describing online information consumption and exposure from an individual user’s perspective, while Sunstein’s echo chamber applies to interaction within a (online) community. As Bruns (2019) puts it, the notion of echo chamber applies to a social network in its entirety, as it comes into being when some users choose to connect with others, shaping up a group and excluding outsiders. The more the network’s borders are sharply distinct, and the more connections are created within it, the more isolated the network is from outsiders and their potentially contrary standpoints. In contrast, filter bubbles are theorized to occur at an individual level. Specifically, the more consistently users consume specific information instead of other and the more they communicate with certain users (who share the same interests and opinions). In turn, it would be more likely that the users’ own views and information will circulate within their network and be confirmed, rather than any view or information from the outside.

To summarize, both these notions share an important assumption, which will be closely examined throughout the course of this paper. Specifically, both these theses share a specific view on the quality of digital technologies’ impact on society. Both echo chambers and filter bubble are seen as a novel dynamic that had a significant impact in society as technologically determined (specifically the filter bubble) or incentivized (specifically echo chambers). So, their most relevant common trait, *would they actually exist as Pariser and Sunstein intended*, would be their being generated and potentiated by digital technologies, independently of the wider societal context where they are introduced.^10^ It is this technological character that turned a great deal of attention from both academia and the general public to the negative ways digital technologies affect daily life.

### Four Problems with the Filter Bubble Thesis: Toward a More Inclusive Understanding

After having introduced the notion of filter bubble and its relation to the notion of echo chamber, we should briefly survey three criticisms that both these ideas faced in the years succeeding their introduction. We will also advance a fourth critical comment regarding these theses, which will give shape to our argument in the rest of the paper, helping us re-thinking the filter bubble and the echo chambers hypothesis as a by-product of a more articulated epistemological situation.

The first amply accepted criticism regards a fundamental aspect of both these notions: the idea of *informational seclusion*. Both Pariser and Sunstein argue that Internet users will tend to find and engage with information and other users that agree with their views. For Pariser especially, such seclusion occurs thanks to the platforms’ algorithms and the way they provide information to individual users. In contrast, for Sunstein such seclusion can be generated both by user choice and by algorithmic recommendation—possibly at the same time—and affects an entire social network of Internet users. However, with the exception of research from Flaxman et al (2016), Quattrociocchi et al. (2016), and Wollabæk et al. (2019), the wide majority of empirical research found very little evidence of algorithmically generated informational seclusion. In other words, people online not only do see, but also engage with pieces of information that oppose their previously and currently held beliefs.^11^

In the case of Google, previous searches and geographical location were not found to affect the search engine’s results in any way when it comes to news and potentially polarizing issues^12^ (Haim et al., 2018; Krafft et al., 2019; Nechushtai and Lewis, 2019; Cardenal et al., 2019). In the case of Facebook, most users seem to find and engage with people of different opinions (Beam and Kosicki, 2014; Beam et al., 2018) and to be exposed to a variety of different standpoints (Bakshy, et al., 2015; Fletcher & Nielsen, 2018a, 2018b; Fletcher et al., 2020). In fact, members of an online community who share certain views might actually seek out other users with divergent beliefs (Smith and Graham, 2019). In the case of Twitter, some politically savvy users were found to be “embubbled” within the ideology of the online community they belonged to, in contrast to more casual users in the same network (Garimella et al., 2018; Williams et al., 2015). However, these online communities are not close-knit: networks of users might cluster around shared interests and topics, but their members are still in contact with other networks (Bruns et al., 2017) and sometimes they actively seek groups with opposing standpoints to engage them in debate (Yardi and boyd, 2010). Finally, some of the studies claiming an occurrence of informational seclusion typical of filter bubbles and echo chambers had somewhat contradictory findings: users were found to be polarized and more likely to conform to preexisting beliefs *and at the same time* to be exposed to opposite standpoints (Flaxman et al., 2016; Quattrociocchi et al., 2016; Wollabæk et al., 2019).^13^ All these studies seem to show that Internet users find and engage with standpoints different from their own, rather than only with content conforming to their views.

The second criticism (which we direct to Pariser’s argument specifically) regards the presuppositions of what kind of information people engage with online, and why. Pariser seems to imply that the wide majority of Internet users will engage in politically oriented searching behavior, making a somewhat strong presupposition on *user’s preexisting attitudes as politically oriented*. The argument presumes that users are mainly motivated by political engagement while using web-based platforms. That is not necessarily the case (Fletcher et al., 2020; Bruns, 2019): actually, people might, more often than not, be incidentally—not intentionally—exposed to politicized information online, while they might mainly use the Internet for other purposes such as entertainment or sociality.

The third criticism is constituted by the general attitude manifested by Pariser and Sunstein toward the social impact of digital technologies, an attitude describable as *technological determinism*. This is the idea that “the technology prevalent in a society will drive the behaviors of its citizens and hence the social structure as well as cultural values” (Dutton et al., 2017) As Bruns (2019) and Fletcher et al. (2020) point out, the inherent problem of the notion of filter bubble—and of echo chamber, to a lesser extent—is the appeal to “purely” technological dynamics to explain and warn against negative or unexpected socio-political developments. They both point out that these terms entered everyday discourse after critical events such as the election of US president Donald Trump and the results of Brexit referendum in England. Bruns argues (2019, 115–121) that the rise to prominence of filter bubbles and echo chambers as explanatory concepts for these political developments might be more of an explanatory shortcut than an accurate account. They would help shift the blame for societal problems to technology, rather than actually understand those problems in their wider context. Moreover, whereas Big Data companies such as Facebook could definitely improve their standards when it comes to the ethical and societal implications of their corporate decisions (Grimmelann, 2014; Cadwalladr, 2018; Lapowsky, 2019), to argue that any political development rises from black-boxed algorithmic dynamics overshadows already present problems at a societal level. The technological determinism assumed by Pariser and Sunstein, in other words, might oversimplify the impact of digital technologies in our society. And if that is the case, then, research trying to understand these dynamics should expect that the relevant processes characterizing the impact of these technologies in everyday life should account more than the effect of algorithms over how people consume information.

Thus, a fourth criticism should be advanced at this point, discussing in detail whether and in what sense the filter bubble and online echo chambers should be considered socio-technical problems, after pondering on the justified accusation of technological determinism that depicts Pariser’s and Sunstein’s approaches. Taken as epistemological arguments (and not as sociological ones), their approach would aim at explaining belief fixation, polarization, and radicalization in social networks with the creation and diffusion of particular technological systems. Without arguing against a correlation between the two events, we argue against the possibility of speaking of causation between the two.

To sustain our position, we can comment on one experiment that tries to consider the relation between technology and their sociocultural context. The experiment was conducted by Davies (2018), who confronted two groups of British high school students from different social classes, in order to investigate whether Google would provide different results depending not just on their previous searches, but also on their level of media literacy and awareness about the research engines. Whereas the participants were relatively few, he did confirm his original hypothesis: the informational embubblement theorized by Pariser occurred only in some cases. In these cases, the embubblement was heavily determined by the persistence of the subjects’ previous beliefs in front of results that seemingly disconfirmed them, which also depended on the subjects’ varying degree of expertise about how to look for information online. In one case, a student was asked “Should we [England] have more or fewer restrictions on immigration?”; and having already the opinion that more restrictions should be made, he typed search terms such as “how much benefits do immigrants get” and “immigrant mansions,” instead of “reasons for immigration” or “immigration economic implications” (p. 649–650). Using different search terms that are supposedly related to the same topic can lead to very different information and perspectives (Borra & Weber, 2012). For this reason, Davies proposed, and we agree, that we should rethink filter bubbles and—we add—online echo chambers as socio-technical problems, whereby not only digital technologies but also pre-existing attitudes constitute an informational embubblement. Thus, if technological determinism falls into a sort of “correlation is causation” fallacy (which mistakes the radicalization and polarization in social media as an effect of the personalization algorithms instead of contemplating much more complex and old-school reasons), then we are left with a question that demands a more encompassing answer. In which kind of relation should we think of the sociocultural intellectual seclusion and the technological one?

Following the idea that an epistemic (and not simply informational) seclusion might be already in the user’s head *before* her engagement with digital technologies, in the next section we will discuss some theories that have been advanced regarding which kind of embubblement can affect people’s attitudes toward the formation of new beliefs; and why at most digital technologies might play a role in either mitigate or radicalize them—in a reflecting, instead of determinist or symbiotic, kind of fashion.^14^

## Epistemic (and Other) Bubbles and Epistemic Discomfort

The possibility that the agent could hold on and share beliefs and positions with unjustified confidence, even arrogance (Tanesini, 2016), has been one of the key topics of contemporary theoretical and social epistemology before the emergence and diffusion of digital technologies (Cassam, 2018). Now the discussion has only an ampler range of applications. In particular, the so-called “bubble theses” (Arfini, 2019) are arguments that aim at discussing how some common cognitive states (such as beliefs, doubts, and moral stances) can both favor the epistemic growth of the human agent and become sources of delusion for her. The theoretical assumptions of the bubble theses are few and simple: a) human beings are fallible agents that fixate beliefs to act into uncertain situations; b) from a phenomenological perspective, believing to know something (which can be seen as fixating a belief) and knowing the same thing appear the same; c) hence, confidence regarding one’s own beliefs is intrinsically highly fallible and may delude the agent on the robustness and legitimacy of her positions. Thus, we turn to review the “bubble theses” because we maintain that they provide an excellent foundation for re-elaborating the concept of filter bubble, by grounding it in the intrinsic cognitive and epistemic limits of human beings, and how these constraints are affected by the interaction with digital platforms, intended as artefacts.^15^ The name and scope of the bubble theses that provide such grounding are the following:

1. The *epistemic bubble*, which has relevant implications when considering how people gain knowledge and feel the need to do so;
2. The *moral bubble*, which affects how people morally evaluate each other’s actions;
3. The *ignorance bubble*, which has an impact on how people estimate their ignorance.

In this part of the article, after describing these situations in detail, we will present how taking them into consideration can be of use also when reflecting on problematic human-to-human interactions in digital environments.

### The Epistemic Bubble: The Categorical Shortcomings of Belief

Woods’s (2005) notion of epistemic bubble originates from a few somewhat truistic considerations: human beings ordinarily make many errors regarding what they know and what they think they know. However, their tendency to formulate accurate beliefs is generally taken to be a relevant contributing factor for the sophistication and success of our species (Clarke, 1990; Griffith and Wilkins, 2010; McKay & Dennett, 2009, Woods, 2005). Indeed, when knowledge is necessary for action, and when the agents are aware that they lack the necessary knowledge to act, they become cognitively irritated^16^ and develop the urge to relieve this irritation by elaborating a belief (Peirce, 2011). The possession of beliefs (in best case scenarios, of knowledge) is in principle pleasurable: agents, by formulating a belief that they think is true (or reliable enough to act upon it), relieve the psychological and practical tension derived from not knowing what to do in particular circumstances. However, this situation creates a phenomenological issue: from the first person perspective the agents do not think they *believe* something, but that they *know* the same thing. Hence, it is impossible for the agent to discern, phenomenologically and in the present moment of the belief formation, whether the agent knows or *simply believes* to know something. Such discernment is possible only in the aftermath of the reliance on the belief, either through the agent’s experience or from other people’s feedback.

Knowledge is, on the one hand, elaborated through beliefs since, of course, knowing something implies believing to know the same thing. However, because belief and actual knowledge are phenomenologically indistinguishable, the formulation of beliefs can actually prevent their verification as knowledge (because the agents already *believe* that they *know*). In this sense, “belief is both a condition of knowledge and an impediment to its attainment” (Woods 2005, 739).

Of course, this embubblement does not make it impossible to gain actual knowledge. Human beings’ openness to third-parties’ feedback, as well as their capacity to gain new information, can support the adjustment of beliefs to reflect more accurately the state of affairs. This, at the same time, does not make the revision of beliefs an escape route from the epistemic bubble. Rather, the continued revision of beliefs is implied as a shift to an epistemic bubble to another. As Woods describes it, even knowing about the epistemic bubble does not make us immune to it: “Although a cognitive agent may well be aware of the bubble thesis [of the structural incapacity to distinguish knowledge and belief] and may accept it as true, the phenomenological structure of cognitive states precludes such awareness as a concomitant feature of our general cognitive awareness” (743).

So, to be aware of one’s own epistemic bubble does not, in principle, support a way to “escape” it. Unless the agents *become aware that* (as they formulate *another belief* about) there is pressuring evidence or trusted social feedback in favor of the contrary, they will maintain that they know what they think they know. In other words, the feedback provided by not just other viewpoints, but also conventions, common sense and social norms is also a condition for furthering knowledge and understanding and at the same time an obstacle to its attainment.

### The Moral bubble: the Systematic (Mis) Representation of Violence

The sociocultural aspects of the epistemic bubble have been especially investigated by Magnani (2011), who specifically reflects on the human limitations in the understanding of violence. Magnani argues that the problem of the epistemic bubble encompasses also moral beliefs, our opinion on what actions qualify as violent (and so as moral or immoral). The ordinary tendency to maintain beliefs and take them as legitimate, highlighted by the epistemic bubble thesis, is even more evident when it comes to moral beliefs (Holt et al., 2009). The experienced legitimacy for maintaining a moral belief is not just motivated by the perceived state of affairs, but also by variables dependent on the social and cultural context of the agents involved (Sommers, 2009). The experience of validity and legitimacy characteristic of moral beliefs is not due to the need to describe a state of affairs in the world, but to the expectation of how the world is supposed to be (Boyd & Richerson, 2001). Magnani points out that moral norms, options, and orientations so constrain our perspectives in exclusion of others: I might have a belief regarding certain practice being violent (and so immoral), but this belief and its sources (such as my education, social class, and moral norms) prevent me from the possibility of unbiasedly evaluating the presumed violence of the target practice.

An example can help clarify the scope and implications of the moral bubble. The veil (*hijab*) worn by Muslim women might be seen by Western atheists or people of Christian descent as symbolic of a form of oppression toward women (Ruby, 2006). However, part of the reason why Muslim women ought to wear a hijab is that this piece of garment is meant to help them avoid harassment and undesired attention from other men (Gabriel & Hannan, 2011). In a case such as this, the very same practice can be seen as a perpetration of violence on the one hand and as the exact opposite on the other: the moral beliefs of each perspective on this norm is diametrically opposed.

Moral beliefs, therefore, constitute what Magnani calls *moral bubbles*.^17^ The notion of a moral bubble shows that our epistemic capacities not only limit our discerning between knowledge and mere belief, but also the discernment between legitimate and illegitimate actions. This discernment is, furthermore, reinforced by, if not outright grounded in, the moral community the subject belongs to: the possession of moral beliefs is greatly determined by the norms and values shared by the community. To follow a system of moral beliefs and values, which can vary across cultures, has a cooperative function and can be seen as evolutionarily advantageous (Sommers, 2009). However, it is at the same time a system that allows violent behaviors through practices of norm enforcement such as punishment, sanctioning or mobbing. It is due to this structural feature that human beings are incapable of recognizing—or they morally justify—the presence of violence in their own moral beliefs (and related moral behaviors).

### The Ignorance Bubble: the Underestimation of the Unknown

Arfini (2019), elaborated what can be called a foil of Woods’s epistemic bubble, that highlights even more the structural limitations of human cognition. Just as human beings are phenomenologically incapable of distinguishing between what they know and what they believe they know, they are also incapable of discerning what they do not know and what they think they do not know. In other words, human beings are naturally prone to underestimate their ignorance, and to think they know what are the things they do not know. If the incapacity to distinguish between knowledge and belief is at the core of the epistemic bubble, the incapacity to distinguish between our actual ignorance and what we think is the extent of our ignorance (our doubt, uncertainties, etc.) is the core of the *ignorance bubble*. And while the ignorance bubble is a complementary aspect of the epistemic bubble, its extent can be considered much wider.^18^

When we are stricken by doubt, by the awareness that we do not know something, we are well aware of it due to the irritation it brings us as cognitive agents, and requires effort and reasoning to solve it. However, doubt only involves the amount of ignorance we are aware of and it is almost physiologically impossible to be aware of the actual extent of our ignorance. Our irritation would make us motionless and incapable of proper everyday action. Author’s reflections point out, in other words, that a cognitive limit has an impact on how exactly we can estimate our ignorance.

Of course, the human tendency to underestimate their ignorance is not a novel research issue in psychology and philosophy of mind. A notorious example is the Dunning–Kruger effect (Dunning and Kruger, 1999, 2002; Dunning 2011): that is, the tendency of people to overestimate their competence when they have little in certain domains. However, while the Dunning–Kruger effect describes cognitive shortcomings of agents in particular circumstances (developing a skill set, for example), the ignorance bubble, just as the epistemic bubble, describes a structural feature of human cognition, that is, in a sense, practically motivated: a lot of the things we do not know are not useful to carrying out our everyday business.^19^ To put it with a blunt example, the knowledge of the diameter of the Earth or of the subatomic composition of Mendelevium is not intuitively handy for a philosophy student. Nor the human brain is computationally capable of processing the entirety of knowledge coming from all the existing scientific disciplines. Ignorance needs to be accepted as a pervasive feature of everyday life, as human beings, the subjects of ignorance and knowledge, are situated beings with situated interests and goals.

The ignorance bubble thesis, however, has some somewhat more disquieting implications than Woods’ original argument. When people engage in their everyday business, while they might feel the pressure to revise what (they think) they know, the same often does not necessarily hold for what they (think they) do not know. The agent’s ignorance is understandable as a massive frame that she cannot help ignoring. The ignorance bubble represents, therefore, a much more extensive form of embubblement than the epistemic bubble, where the success of the agent’s everyday and situated practices impedes her from realizing the extent of her ignorance and from questioning how much she does (not) know.

### The emotional Side of the Bubbles:Eepistemic Feelings and Discomfort

A stable feature of all these bubbles is their practical function in the everyday experience of the agents, that allows them to believe they have knowledge at their hands, to feel that they are not behaving violently, and to avoid feeling crushed by the depth of their ignorance. At the same time, what allows us to discuss and describe the function of these bubbles is the fact that in some circumstances agents do need to confront the fact that their knowledge is not as sound as they thought, their behavior might be not morally accepted by others, and their ignorance is much deeper than they predicted. In these cases, what has an impact on the agents is not just the epistemic or moral acknowledgement of a situation, but also related emotional responses which are studied in metacognitive literature with the term *epistemic feelings*.

Epistemic feelings are elements of the emotional spectrum (Arango-Muñoz, 2014) with meta-cognitive functions and which affect cognitive abilities, states, and decision making (Sousa, 2009; Evans, 2008; Terpe, 2016). Examples of epistemic feelings that we experience in everyday situations are the feeling of knowing or the tip-on-on-the-tongue feeling. Doubt, in this sense, is irritant (also called epistemic anxiety in psychological literature—Hookway, 1998), because it is the acknowledgment of one’s own ignorance. As we already discussed, when we experience doubt we want this sensation to end as soon as possible: agents can end this state of irritation by acknowledging the doubt for what it is, and by making the cognitive effort to get to know the state of affairs that is the source of doubt. However, sometimes making this effort is not feasible, either due to a lack of previous information or due to a lack of motivation, and agents might simply downplay their ignorance in order to avoid the irritation brought about by the awareness of it. The Dunning–Kruger effect is an instance of the way human beings devalue, often unintentionally, the width of their own ignorance. And the downplaying of one’s own ignorance is not unmotivated, nor intrinsically vicious.

For the purpose of this paper, we will highlight the experience of not-(yet)-acknowledged doubt: the situation in which people believe to know something instead of actually knowing it, and they actively try to avoid situations that challenge that belief. We argue that this state is not emotionally neutral: indeed, we described the act of forming a belief as a relief with respect to the state of doubt. But after the relief passed, it would be consistent with literature on emotional cognition to say that maintaining a certain belief would not be just a peaceful circumstance (Moore & Oaksford, 2002), especially if the agents are vaguely aware that they have, in a way, downplayed their ignorance to feel confident in believing something. We call the epistemic feeling agents have when they interact with the possibility of having one of their beliefs actively challenged, or of finding out that they do not know something, *epistemic discomfort*. Of course, epistemic discomfort is phenomenologically akin to doubt. However, and very importantly, doubt implies an acknowledgment of one’s own ignorance; in contrast, epistemic discomfort does not imply such awareness, as the person needs simply to engage with the possibility that their beliefs are false, and not actively entertain with the idea that they are false.

Before discussing how epistemic discomfort may emerge as a consequence of embubblement or informational seclusion online, we think it might be useful to provide a brief vignetta to present how epistemic discomfort may also emerge in offline contexts. The scenario will be useful to clarify exactly what we mean with these terms and to specify that we do not think they represent only online-based occurrences. So, let us consider the following example. Anna, who lives in a small, isolated, and staunch religious community, is walking down the street, when a man, Bob, comes to her to ask for directions. While talking back to him, Anna notices some characteristics about Bob: he’s wearing some eyeliner and nail polish; his midriff is slightly exposed; and his voice is slightly high-pitched. Anna possesses a somewhat traditional conception of masculinity, and has never met an openly homosexual man beforehand. In this scenario, she might feel uncomfortable about her brief encounter with Bob, because she met a man whose apparent characteristics do not conform with her conception of masculinity—so, *what she thinks she knows* about what men are and how they behave. This encounter implicitly (and unwillingly) challenges her assumptions about masculinity because Bob does not conform to that conception; however, that does not mean that she *doubts* the validity of her conception of masculinity. She does not know that her conception of masculinity does not account for instances that contradict it. In other words, she is ignorant about the lack of universal appropriateness of her beliefs about masculinity. However, she does not doubt the validity of her beliefs, because she does not acknowledge that they might be wrong, and that she might actually be ignorant about what masculinity is or what is supposed to be. The experience of this unacknowledged contradiction causes *not just irritation*, but *discomfort*, because it hints at the possibility that her beliefs are inadequate to account for the designated state of affairs.^20^

So, in case of epistemic discomfort, which are its practical consequences? Anna’s encounter with Bob, while implicitly clashing with her beliefs, does not necessarily provide sufficient grounds or motivation for her actually questioning the validity of those beliefs. Instead of questioning the validity of her beliefs about masculinity, she might downplay or deemphasize Bob as an exceptional or aberrant case. Of course, in other circumstances, epistemic discomfort might cause the formation of a doubt; but it might, in others, even strengthen the agent’s (inadequate) beliefs instead of pushing her to question their validity. In those cases, epistemic discomfort might so reinforce the agents’ ignorance and epistemic bubbles instead of pushing her to change them.

In the next section, we are going to analyze some concrete settings where, we suggest, this dynamic actually occurs: social media. We will argue that the filter bubble should not be understood as a purely algorithmically driven mechanism, but as the result of epistemic discomfort, caused by the way users of social media interact with the beliefs and moral assessments of other people. The reason for this, we maintain, is the way such beliefs and assessments are conveyed to social media users through the platform.

## Rethinking Filter Bubbles and Echo Chambers: Phenomenology of Belief and Social Feedback on the Web

We will now proceed to reformulate the notion of filter bubble and echo chambers in light of the bubble theses we examined so far. Specifically, we will argue that the filter bubble and echo chambers should be understood as conflations of the epistemic, moral, and ignorance bubbles of Internet users with the way information is configured within the digital platforms they use. The filter bubble and online echo chambers, we argue, are not exclusively generated by recommendation systems, but by the way information and other users’ beliefs are presented to one another by the platforms. In other words, it does not matter *what* is presenting the information to people online (i.e., the preference algorithms), but rather the way, or *how* such information is presented to and experienced by the people that interact with it online. The filter bubble and online echo chambers, in other words, are not phenomena purely related to algorithms and what information they present, but to how people react to and interact with information that algorithms present them with—it is not about the information in itself but to people’s relation to the information presented on the platform. When the users encounter information discordant with their previous (embubbled) beliefs, it can cause, we argue, epistemic discomfort, which will tend to push users to stick to their own beliefs and reinforce their epistemic bubble. This is especially true in social networking sites (henceforth SNSs) such as Facebook or Twitter, due to their focus on interpersonal relationships (Cheung & Lee, 2010), which enables people holding contrasting beliefs to interact with opposite standpoints in a *non-mediated way* (as we will discuss further later on). For this reason, we are going to primarily focus our analysis on SNSs.

The argument will proceed as follows. Firstly, we are going to provide a perspicuous representation of digital platforms as environments constituted by information. We will note that even other people are presented to others as sums of the information they provide to the platforms and the content they generate, and that the user’s interface of SNSs stands in tension between privacy (as the interface is their own) and public (as other people and sources can access it and interact with it). Secondly, we will build on this characterization of online environments to argue that people might tend to conform and seek standpoints confirming their own opinion due to the unmediated and sometimes unexpected way contrasting standpoints interact. In sum, we argue that epistemic bubbles have a different impact online and offline: in SNSs, the unmediated nature of the interaction fosters epistemic discomfort instead of favoring the emergence of doubt, making people more rigid when they encounter contrasting opinions. We will then support our argument by re-interpreting ambiguous results of studies claiming to have found evidence of filter bubbles and echo chambers, and we will argue that online embubblement, as well as the creation of echo chambers, should be understood as *deliberate*, i.e., as a result of the choices of users, and not purely caused by recommendation systems.

### The Materiality of Others and of Information on the Web

Understandably, SNSs are of special interest for philosophy of mind and epistemology because, from the user’s perspective, “there is no gap between information and matter” (Arfini et al. 2019, 382). Digital environments are performed by human and artificial agents^21^ that provide users with information, which is primarily (but not exclusively) about people, institutions, and events that exist in the offline world. What Internet users see, however, is just information: while offline in many cases information is situated (socially, culturally, normatively—Cobb, 2001) and mediated by various elements of the context in which people interact, in online platforms users *directly* engage with information without the mediation of a shared and embedding context. While philosophical approaches to cognition that take into account the influence of the environment are not new (see Bateson, 2000; Clark, 2008; Malafouris, 2013; Hutchins, 2010, 1995; Menary, 2010), web-based platforms represent an exceptional instance of cognitive ecology (Smart et al., 2017a, 2017b). Not only are they of exceptional interest because they are constituted by information, but they also afford a very wide range of activities, forms of interaction with information and other users, and allow for varying degrees of personalization.

As Bertolotti et al. (2018) point out, Internet users themselves (and, from an individual user’s view, other people online) are configured as bundles of information. Building up on Waite and Bourke (2015), they argue that the association of users of digital platforms with the platform—especially in the case of SNSs—constitutes a case of “cyborgification”: the human user and the used piece of technology are coupled in such a way that the artifact co-constitutes the way the user cognizes and experiences the world (Clark, 2003; Verbeek, 2008, 2011). While the use of an artifact that grants Internet access does not make an agent a cyborg, the same does not hold when we consider the user from within the platform. At the level of abstraction of SNSs—that is, when considering users as part of the interface of SNSs—users are inseparable from the content they produce and the information they provide. The profile settings and information they set up, their network of “friends” online, the information sources they follow and the content they generate—their opinions and expressed values—are what constitutes a user from the perspective of the platform, both in the ways they can act in the platform and in the way they are perceived by others on the platform.

This characterization of digital environments is meant to highlight what might be called the “merely” informational quality of digital platforms. To interact not just with digital platforms, but even with other people on the platforms, means to interact with information—which is structured by the platform’s algorithms and provided by other people and agents on the website. While this kind of consideration is not particularly novel, what is relatively unexplored is the way this “merely informational” status of other people, institutions, and information sources affects users’ experience of those sources. Due to the meshing of agents and the information they generate on these platforms, one can say that, online, we see other people’s beliefs *before* the people themselves. A platform such as Facebook is designed so that its users define themselves based on their beliefs, opinions, desires, preferences, and values—ultimately, based on the content they generate, the sources they follow and the traces they leave on the platform.

Furthermore, the consideration of digital environments as merely informational helps highlight a fundamental ambiguity of many web-based platforms (and, most prominently, social media such as Facebook and Twitter). This ambiguity can be defined as the blurring between private and public space. Consider the case of Facebook’s newsfeed, “the constantly updating list of stories in the middle of your home page [which] includes status updates, photos, videos, links, app activity and likes from people, pages and groups that you follow on Facebook” (Facebook, 2021). Through the newsfeed, the information on the platform is perceived and understood from the user’s perspective as her own. However, while the experience and structure of these platforms is user-oriented and personalizable, whatever content one produces and consumes also exists in an interpersonal dimension: in sum, from the user’s perspective, other agents “fade” into the background of the platform. They are perceived as part of the informational environment of these websites, and they become objectified and undifferentiated as bits of information.

It is both the natural limitations of computer-mediated communication and the design choices of these platforms (the constitution of a profile and how it explicitly invites you to post what is on your mind) that lead users to consider not only each other, but eventually also themselves, as their generated content. “Online, it is often easier to separate people from their embodied experiences, or to mistake the part for the whole—or to never even see the whole, and therefore never understand the context from which a particular collection of pixels has been unmoored” (Phillips and Milner, 2018, 89). The settings and structure of the platform leads to the understanding of other agents online only based on what they post and share, ignoring that they are people grounded in an offline—and unseen—context and merged with a platform that transforms their experience and cognition. Or, as Nelson (2018) puts it: “Social media allows us to persistently emphasize who we are and set aside the question of what we are altogether” (178).

### Context Collapse and the Unmediated Experience of Information Online

We now turn to the analysis of what we have called the “immediacy” or “unmediatedness” that characterizes the way users experience information in many different platforms, and mainstream SNSs most prominently. The specificity of this immediacy originates in an issue intrinsic to computer-mediated communication already individuated by Kiesler et al. (1984). They wrote: “[c]ommunicators must imagine their audience, for at a terminal it almost seems as though the computer itself is the audience” (p. 1125). One of the most trivial differences between online and face-to-face interaction is the fact that the computer user does not *see* the recipient of her communicative acts. While through media such as email or private instant messages the perception of the message recipient can be relieved by the knowledge of who is going to consume that information (i.e., you know who you are messaging) the problem of imagining who is going to consume the information you produce is prominent in platforms such as SNSs. For in these platforms, the content users consume, share, and produce is potentially shared to and by the entirety of their online social network, which generally includes people from very different offline social contexts—your friends, your co-workers, your family members, and mere acquaintances to name a few. Even strangers, under certain circumstances, can come into contact with your posts, comments, and reactions online. This feature of many contemporary forms of computer-mediated interaction and communication is known as context collapse: “[T]he flattening out of multiple distinct audiences in one’s social network, such that people from different contexts become part of a singular group of message recipients” (Vitak, 2012, p. 451).

The concept gained much attention since its introduction by danah boyd (2008; Marwick and boyd, 2011; Gil-Lopez et al., 2018) to describe how the indeterminacy of the audience on SNSs such as Facebook and Twitter leads users to moderate content production and self-presentation. Because people do not really know who is going to actually consume the content and how would they react, many Twitter users feel the need to imagine what is appropriate to post to match the expectations of all possible content consumers—friends, family members, strangers, and so on. However, while context collapse has been generally studied in relation to privacy management (Vitak, 2012; Marwick and boyd, 2014) and exposure to news online (Beam et al., 2018; Kim & Ihm, 2020), there is still some uncertainty in the literature regarding its exact conceptualization and implications (Davis & Jurgenson, 2014; Costa, 2018; Szabla and Bloemmert, 2020).

For the purposes of this paper, context collapse is relevant because it constitutes the source of what we called the *immediacy* or unmediatedness of information online. Specifically, the immediacy of information is determined by context collapse because the merging of different social groups leads to the indetermination of normative standards for communication and interaction with others. The impact of context collapse in online self-presentation is due to the fact that different (offline) social groups have different expectations for what it is appropriate to say or not to say. The various expectations, more or less acknowledged standards for appropriate and inappropriate behavior that underlie social interaction in different face-to-face settings become undetermined. The audience of the content you produce sets normative standards for how you choose to behave and what you say. While you would behave differently in front of strangers and with friends or family members, online all of these audiences, as consumers of the content you produce, are reduced to one. Online, expectations regarding the consumption and origin of content, as well as interaction with others, becomes indeterminate. The information consumed on SNSs is unmediated because there are not universally shared norms for interaction that can help framing and interpret that information univocally—a lack of frame that can cause an experience of that information as unexpected, unmotivated, or even unjustified.

### Reinforcing Epistemic, Moral, and Ignorance Bubble online

So, let us refocus on the impact of the bubble theses if we take into consideration the merely informational nature of digital platforms from the phenomenological perspective of users. Let us consider the following scenario. Andy and Betty are two Facebook users who are on the platform to consume the news. Andy firmly believes that the vaccine against COVID-19 is safe and effective: he is aware of the wide amount of misinformation and conspiracy theories out there, and he firmly believes that scientific research cannot (epistemic belief) and should not (moral belief) be disputed from non-scientific perspectives, such as politicians or religious institutions. Betty, on the other hand, is not as sure about the safety and effectiveness of the vaccine: she knows that online there is much contradictory information on the matter, and she does not necessarily have a strong opinion regarding which sources have a higher degree of authority on the matter. Suppose that, while on Facebook, they independently stumble on the same post: for instance, a post with a hyperlinked article that mentions how a religious institution deems the Johnson & Johnson vaccine dangerous (epistemic information) and immoral (moral information). Andy swiftly writes, in the comment section of the post, that in no way a religious institution is entitled to deem the validity (epistemic bubble) or morality of a scientific practice (moral bubble), and that he’s appalled by people who ignore or do not just follow the scientific consensus in this regard. Betty, in contrast, reads not just the post, but also Andy’s comment. She feels uncomfortable and offended by this comment: she does not “just follow” the scientific consensus, as her feelings and beliefs about the vaccine also come from her social group and, in general, sources that she deems reliable (epistemic bubble) and just (moral bubble), which Andy does not consider. She therefore comments as well: she calls out Andy for his ignorance regarding other people’s sources and questions his proclaimed entitlement regarding who can express her opinion about what. After this brief interaction, Andy will feel even more sure about his outstanding opinion regarding vaccines, as his experience of the alternative view was still negative (reinforcing both his epistemic and moral bubbles); while Betty will feel even more insecure regarding vaccines, as she experienced the interaction with alternative view(s), as violent and wrong (reinforcing her bubbles, as well).

This short story regarding a non-constructive interaction between two social media users is a paradigmatic case that stitches together all the various conceptual threads we presented in the course of the paper. In particular, this interaction constitutes an example of epistemic discomfort in online settings: if we take the perspective of either Andy or Betty, what we see is an agent who is confronted with someone else’s perspective that questions the veracity of her epistemic and moral bubble. The “evidence” the agent experiences challenges her and pushes her to consider her ignorance on the matter at hand—here, on the safety and morality of vaccines. The agent, here, does not experience doubt: s/he does not acknowledge the possibility that s/he might, in fact, be ignorant and wrong on the matter at hand. In a sense, there are not even sufficient grounds for his/her to do so: s/he is just told by another person that she is wrong about it.^22^ In this case, epistemic discomfort does not lead to a reconsideration of the agent’s views, but to their confirmation—to a reinforcement of his/her epistemic and moral bubbles.

This dynamic is enabled by the specific conditions of online environments. As we have pointed out, in digital platforms everything the Internet user sees is information, even in the case of other people. While Betty cannot see or know about Andy’s frustration regarding misinformation on COVID-19 online, Andy cannot know or predict how Betty (or other people) consider a source reliable and their motivations to do so. Andy only sees a Facebook post that he considers (epistemically and morally) wrong, and is appalled that other people do not agree with him; and Betty only sees someone who criticizes anyone who disagrees with his ideas. The contrary perspective is experienced as unmediated, decontextualized and unjustified in tone and scope; the way it is encountered—which, it must be noted, arises from the way the platform is designed and configured—causes epistemic discomfort that is not yet sufficient for the explicit formulation of doubt. Therefore, Betty (or Andy) can choose to discredit the validity of the contrary perspective (or, as Nguyen [2020] puts it, epistemically discrediting it).

The confrontation between agents or information sources with contrasting perspectives and opinions leads them to perceive unmediated clashes between opposite and almost incompatible views (so epistemic and moral bubbles). The immediacy of this clash can lead to the reinforcement of the epistemic bubble instead than to an open reflection of one’s own (possible) ignorance. This reinforcement, if offline would not have been sufficiently motivated, if considered within online environments should not be necessarily described as irrational. Due to context collapse, the merging of different expectations for what is appropriate to say, share, or behave that are grounded in different offline situations contributes to a generalized uncertainty regarding how to react in front of unexpected situations. As Rini (2017) points out, on the contrary of face-to-face interaction, norms of communication on social media are disputed: the aims, purposes, and causes of a specific post are not undisputedly understood by social media users. Users can only assess other people by what they actually post; they lack both access to other users’ motivations and background, as well as a shared understanding of norms of communication and behavior. In this context, taking an epistemically partisan stance toward contrary opinions—that is, reinforcing one’s own previous belief in front of contrary standpoints—can be considered as a rational choice. Because the opinions and claims of other people online come to be in an underdetermined context, sticking to one’s own perspective can provide interpretative support of information and claims generated from others. It does so by implicitly establishing who is an epistemic and moral peer and who is not and by elucidating one’s own values. When one experiences epistemic discomfort online by encountering unmediated and seemingly unjustified opinions (a feeling that may be expressed with the words: “How can you say that?”) reinforcing one’s own epistemic, ignorance, and moral bubbles can be seen as the most intuitive strategy.^23^

### Redefining Filter Bubbles and the Arbitrariness of Epistemic Isolation

We now have all the resources to re-formulate the notion of filter bubble by changing the focus of the original problem and taking into account the empirical evidence available regarding information exposure online. Pariser understood the filter bubble as a form of technologically caused informational and epistemic seclusion, where contrary perspectives are excluded by the preference algorithms. In contrast, we reflected on the phenomenology of belief and of experiencing contrary perspectives, and we looked at the transformation of this experience in online environments. We proposed that the experience of epistemic discomfort in web-based platforms is especially due to the way others’ opinions are framed and presented. The unmediated manner other opinions are experienced and how easily they can cause epistemic discomfort in digital environments more reasonably lead to sticking to and maintaining one’s own outstanding opinion than to consciously doubting it, because in the user’s perspective there are not sufficient grounds for doubt.

We therefore define the filter bubble as *the reinforcement of one’s epistemic, moral, and ignorance bubble caused by epistemic discomfort experienced online*. We do not see the filter bubble as caused purely by technology, but by the interaction between user and technology, and specifically by the way users experience each other’s views in online settings. The unmediated way contrary perspectives are encountered thanks to context collapse lead to an uncomfortable experience, as the user’s outstanding belief is questioned. However, due to the indeterminacy and ambiguity of the conditions surrounding the contrary opinion, users will more probably feel prompted to stick to their outstanding opinion instead of doubting it: when the options are either to assume one’s own opinion is correct or to side with a standpoint that questions her own without apparent grounds, that can even be considered reasonable, despite its being irrational.

This technologically mediated experience of epistemic discomfort leads, we suspect, to a transformation in attitude toward one’s own beliefs and the perspective of others. Your outstanding belief, which is being challenged, instead of being the object of your evaluation, it might assume a guiding function toward your evaluation of others’ perspectives, and end up taking a normative role. Because of the uncertainty regarding interpreting and evaluating information online as valid, authoritative or justified, when experiencing epistemic discomfort one can rationally solve it by appealing to one’s outstanding belief as valid—even though it might not be necessarily so. Exposure to contrary or diverse perspectives in these settings can lead not to open-mindedness, but to reinforcing one’s own both epistemic and moral self-righteousness—because one’s own outstanding beliefs become the only reference points while navigating online environments. In this climate of informational uncertainty, to follow one’s own (presumed) knowledge can be considered the most rational—and definitely the least unpleasant—course of action. Unmediated, and eventually unintentional, exposure to contrary perspective would not lead to open-mindedness, but to what Nguyen (2020) calls a “disagreement-reinforcement mechanism” (147): the existence of contrary perspectives can lead to the perceived corroboration of one’s own outstanding belief, especially when the believer expects that other people will attempt to contradict her.^24^ One’s own epistemic bubble is, in this scenario, what supports the filtering of information and the reference point for evaluating other data and others’ perspectives. In this sense, one might say that the filter bubble is “already in your head”—your epistemic bubble will be reinforced by the experience of epistemic discomfort online and not just remain unquestioned, but drive information consumption behavior.

At this point, it must be noted that we are not arguing that all Internet users are going to experience epistemic discomfort in the same way. It is a truism to say that some people are more open-minded than others (and that would probably remain true also in online environments). However, we assume that it is specifically the way most social media users feel challenged online that prompts them to conform to and reinforce their outstanding beliefs. In other words, exposure to contrary perspectives—in a decontextualized, unmediated, and distorted manner typical of most web-based platforms—makes one’s own epistemic bubbles to be more rigid, and to filter out contradicting opinions. Not informational seclusion, we argue, but the way individual users manage unmediated contrasting opinions leads to the cognitive rigidity and tendential polarization that Pariser and Sunstein worry about.

We now turn to test our argument by looking at a group of studies that found some empirical evidence of algorithmically caused informational seclusion, either in the form of filter bubbles or of echo chambers. As Bruns (2019) points out, we should remember that not only the filter bubble is generally understood as algorithmically generated, but even echo chambers are sometimes seen as indirectly caused by preference algorithms. We intend to re-interpret the findings of these studies taking into account also the way users feel about (contrary) information they find online.

Flaxman et al. (2016) analyzed the behavior of more than 50.000 Internet users’ news consumption on 100 online news platforms. They were specifically researching whether the consumption of news provided via social media and web search would have been associated with a higher degree of ideological segregation (of a lesser degree of contact with contrary information) than directly visiting a news website’s page. They did find that social media and web search was indeed (slightly) associated with ideological segregation and information consumption of like-minded news. However, the ideological segregation they found generally reflected offline patterns of news consumption and social media and web search were also associated with *exposure* to a higher variety of outlets than direct search did. In other words, while people are more exposed to cross-cutting news outlets when using social media and web search (in contrast to visiting the outlet’s web page directly), they seem to engage with such cross-cutting outlets less. As the authors put it: “[A]rticles found via social media or web-search engines are indeed associated with higher ideological segregation than those an individual reads by directly visiting news sites. However, we also found, somewhat counterintuitively, that these channels are associated with greater exposure to opposing perspectives'' (318).

This finding seems to question the notion of filter bubble as an algorithmically generated informational seclusion. After all, users of web search engines and social media are exposed to contrary perspectives, contradicting Pariser and Sunstein; however, they do not engage with those outlets despite the fact that they are exposed to them. Our account of filter bubble, which shifts the attention from the “mere” platform to the user’s cognitive processes, can explain Flaxman et al.’s findings as follows: incidental exposure to contradictory perspectives, which will be generally perceived as groundless, causes epistemic discomfort, a reinforcement of one’s own outstanding beliefs, and, ultimately, to the inclination to consume like-minded information. It is, in other words, in virtue of unmediated exposure to contrary perspectives that Web users become ideologically segregated.

The same dynamic of reinforcement of one’s own outstanding beliefs in face of unmediated contrary information is also helpful in explaining the formation of echo chambers. As we saw in Section 2.3, one of the problems with the studies that claimed to have found evidence for echo chambers (Quattrociocchi et al., 2016; Del Vicario et al., 2016; Wollabæk et al., 2019) is the presumption that the online community that constitutes it is very much isolated from contrary information sources. This is actually a pretty heavy assumption: a higher degree of activity with like-minded individuals does not entail that users do not get in contact *at all* with contrary perspectives—at least not on mainstream websites such as Facebook and Twitter. Therefore, one could interpret the notion of echo chamber in a “softer” way, as a quantitative difference in interaction with like-minded vs. contrary users and information sources. This “softer” interpretation of the notion, however, needs to still explain the effect of the outgroup’s opinions and perspectives on individual members of the echo chamber, in light of the fact that digital platforms users are exposed to them.

Our understanding of the filter bubble, as the result of and manner of avoiding epistemic discomfort in online settings, can account for the formation of echo chambers and their maintenance in spite of exposure to contrary perspectives. The experience of unmediated opinions that contradict our own can lead to reinforcing our outstanding beliefs and, ultimately, to cognitive rigidity in front of contrary information. And while research that investigates echo chambers agrees that their members are driven to conformity by a confirmation bias, the previous experience and (mis)management of epistemic discomfort, as well as the desire to avoid it in future digital interaction explain why such bias would develop in the first place—even in agents who were not polarized to begin with. The desire to avoid epistemic discomfort and filter out contrary information can then lead to conformity to whatever information source will confirm and conform to our outstanding belief, including other people who share our beliefs, views and attitudes. In his original essay (2005) on the notion of epistemic bubble, Woods noted that the epistemically closer our source of feedback is, the least can our epistemic bubble burst. In online environments, where information from diverse sources appears qualitatively undistinguishable, seeking interaction with like-minded individuals can reinforce one’s own epistemic bubble and drive information evaluation and consumption.

Our re-elaboration of the filter bubble has a further implication, which stands in stark contrast with the central worries that Pariser and Sunstein underlined. The generalized sentiment that pervades their understanding of filter bubbles and echo chambers consists in the fear that digital technologies will cause intellectual isolation and, therefore, polarization. Unhealthy information consumption and hostility toward contrary perspectives would be explained by the fact that the platform does not allow users to get in contact with different perspectives—a dynamic that empirical evidence that does not presuppose the existence of echo chambers seems to substantially prove wrong. In contrast, our re-formulation of the notion of filter bubble, by taking a step back and looking at how a user feels in the interaction with web-generated information, understands the influence of digital technologies in quite a different way. While it is true that online platforms such as social media enable a high degree of personalization in information management, we argue that it is the modality of experience of contrary perspectives online that causes the reinforcement of one’s outstanding beliefs. It is the unmediated way opposite viewpoints are experienced that leads to conformity and to the discrimination of those perspectives. In this sense, exposure to contrary perspectives in online settings is actually functional to the creation of filter bubbles and echo chambers. The reason being that such exposure is unmediated and often perceived as “out of the blue,” as groundless and unjustified: encountering opposing views in online settings causes epistemic discomfort that leads to rigidity more than open-mindedness. The formation of echo chambers and the individual development of cognitive rigidity is, in other words, the result of users’ choice, not of the algorithm’s decision.

## Concluding Thoughts

In contrast to previous conceptions of the influence of digital technologies on everyday epistemic practices, our interpretation of the filter bubble has some important theoretical and practical advantages. Firstly, describing the filter bubble as the result of user deliberation (encouraged by digital design) caused by epistemic discomfort does not rely on any form of technological determinism.^25^ Instead of attributing the formation of polarized (and potentially secluded) users and communities on black-boxed algorithmic systems, we examine the relationship between the information presented on digital platforms and the belief-formation and epistemic feelings of the user. This shift of focus distributes the responsibility for epistemic rigidity and polarization in between all parties involved in a more nuanced manner. Secondly, our elaboration of the filter bubble as the primary cause of polarization and rigidity is coherent with already existing empirical research that investigates information exposure and consumption behavior, much more so than the original filter bubble thesis and the idea of algorithmically generated echo chambers do. Thirdly, our argument can account for different motivations behind users’ information management online. On the one hand, epistemic discomfort online can also be managed with a higher degree of open-mindedness (even though we take it that that would be the exception more than the norm). On the other hand, the kind of cognitive rigidity that users may feel online and the desire to avoid it is shared by both politically savvy users who use these platforms primarily to seek political information, and those who use them for other reasons and simply stumble upon information. Fourthly, our account identifies a causal relation, instead of a mere correlation, between information consumption behavior in online settings and the formation of a polarized and partisan attitude toward contrary perspectives. And finally, our account can be put into test through empirical research, as the experience of epistemic discomfort and the way it is managed can be explored among social media users in general through qualitative research.

Of course, still relevant questions and issues pertain to the idea of a filter bubble as so reframed. One problem over all is practical: if users in digital environments are prone to reinforce their extremely personal and cognitively relevant epistemic, ignorance, and moral bubbles, finding a way to break this mechanism would involve both changes in the interface’s design and in the ways users will interact with it. On the one hand, the implementation of media literacy policies that can support users’ understanding of how these technologies work and of their own situatedness (and intrinsically limited perspective) within these platforms would be more than adequate—even though such media literacy might be radically different than for traditional media (Phillips & Milner, 2021). On the other hand, however, we believe that the most effective way of tackling the problem of filter bubbles, intended as epistemic discomfort experienced by people on digital platforms, would be to implement some design changes to the interface of these platforms. Specifically, because context collapse and the interpretative uncertainties it implies are one of the primary factors contributing to epistemic discomfort, through the implementation of designed norms it would be possible to contextualize the information people run into online. Such norms may not only help users keep in mind that their personal expectations might be inadequate for the content they encounter, but actually support a more open mind and higher degree of understanding toward others—not only helping frame content that appears groundless, but actively encouraging a more constructive attitude toward the information they encounter.

That being said, while we do think that changes in the design’s interface that tackle context collapse would be the most efficient solution, we do not think of it as the only possible one. We are in good support of a plurality of strategies for making people more intellectually humile, or more open-minded—including not only changes in the interface, but also media literacy policies with that purpose. Eventual changes in the interface’s design to reduce epistemic discomfort will also need to be not conflicting too strongly with the service provider’s corporate interests. Thus, solving this specific problem will probably require the implementation of socio-technological ways to encourage a more constructive epistemic interaction on digital platforms. Given the complexity of this follow-up problem, we look forward to actively exploring its implications in future research.

## Footnotes

1 A terminological clarification here might be necessary. With “the fixation of beliefs” we do not intend to refer to obsessive or irrational attachment to particular ideas. Instead, we, as the authors we will refer to, follow the pragmatic tradition, opened by Charles Sanders Peirce, which presents the state of belief as, simply, a stronger description of belief with respect to the one adopted by commonsense: “[the belief] is a calm and satisfactory state which we do not wish to avoid, or to change to a belief in anything else.” (Peirce, 2011, p. 41). In this account, people fixate on some beliefs because “we cling tenaciously, not merely to believing, but to believing just what we do believe.” (p. 41)

2 We acknowledge that, in a recent publication, Nguyen (2020) utilizes the notion of epistemic bubble to reflect on the formation of echo chambers online and on information consumption behavior. We want to remark here that the two notions are very different: while Woods’ notion intends to capture a phenomenological and structural character of having beliefs (which is not a psychological phenomenon but may have psychological consequences), Nguyen uses this notion to refer to “a social-epistemic structure which has inadequate coverage through a process of exclusion by omission” (143). Nguyen’s notion is closer to our notion of “merely informational environments” (see sec. 4.1, p. 19) as spaces that enable highly personalized ways of managing information and possibly (not necessarily) excluding or omitting other perspectives.

3 Importantly, we acknowledge that the overall mechanisms of digital platforms as business models are mediated by the service providers’ intention to make a profit; however, this is not the relevant sense in which we talk about (un)mediation. Firstly, while we realise the importance of the capitalist logic of the service providers in the design of digital platforms, for the purpose of our paper we are focusing on the relation between individuals and artefacts, and how this relation transforms the individual’s cognitive processing (see e.g. Clark and Chalmers 1998; Clark 2003, 2008; Verbeek 2008; Clowes 2015; Smart 2017; Ihde and Malafouris 2019; Baber, Chemero and Hall 2019; Rosenberg 2019). Secondly, while we acknowledge that the transformative impact of these technologies on cognitive processing can be understood as a form of mediation, we are not following Ihde’s (1990) or Verbeek’s (2005, 2011) theory of technological mediation specifically. With the word “immediacy” and “unmediated” we are specifically talking about a manner of experiencing information in online environments that is determined by some characteristics of digital platforms - most prominently, context collapse. We examine the meaning of this immediacy in detail in section 4.2.

4 See footnote 1 at page 2 of this article.

5 It is relevant to note, as Bruns (2019) points out, that while there is a generalised understanding in the literature of what a filter bubble is, Pariser (2011) does not provide a univocal definition, and definitions vary slightly across publications of different authors. The same occurs with Sunstein's notion of “echo chamber” that we introduce in section 2.2.

6 At best, preference algorithms will provide content similar to that previously consumed.

7 It is worth noting that one of the aims of preference algorithms is what is called online profiling, the recording of online behavior of internet users in order to promote product advertisement. There is a somewhat consistent literature on how profiling constitutes a harm to personal autonomy (see Hildebrandt 2008; Büchi et al., 2019).

8 It is appropriate, here, to make some terminological distinctions. With the term *social network*, we mean to describe a group of social actors interacting with each other, either offline or online. With the term *social networking site*, we mean to describe online platforms (such as Facebook or Twitter) where users interact with each other - in other words, digital platforms where social networks can take form. With the term *online communities*, we mean social networks that specifically form in digital platforms - groups of actors interacting with each other online. To further clarify: for Pariser, filter bubbles are not social networks, but the way the platform’s algorithms affect an individual user’s chances to encounter information. In contrast, echo chambers are social networks which are affected by either the choice of each individual within the network or by algorithmically generated informational seclusion (see section 2.3). Along with the concept of filter bubble, in the remainder of the paper we criticise the existence of echo chambers that are (allegedly) created through algorithmic choice and not users’ choice.

9 For instance, a group of people who would consume television based news from one channel only; or a physically isolated community of people who share the same set of views on a certain topic (e.g. all of whom are Catholic) are examples of offline echo chambers.

10 To be clear, as we will argue in the next section, we think this sentiment, shared by Pariser and Sunstein, is fundamentally misguided, for it assumes a form of technological determinism that we do* not* endorse.

11 It is worth pointing out that the wide majority of these studies, with the partial exception of Fletcher et al. (2020) only considered WEIRD (Western, Educated, Industrialized, Rich and Democratic) countries, and are not necessarily generalizable to the entirety of Internet users around the globe. However, neither Pariser nor Sunstein seem to consider non-WEIRD countries in their theories. More in general, this collection of studies we mention does not aim to prove these theses wrong beyond reasonable doubt, but more simply to question certain aspects that characterize them and might be changed.

12 This is not to say that localisation does not affect Google searches at all, a fact there is clear evidence for (Ochigame and Ye 2021). When a user activates geolocalisation on their devices and access to those data is granted to the search engines installed in those devices, at least some of the sources will be affected by geolocalisation. For instance, Google results for the term “God” will vary depending on the location of the user. Similarly, searching for a term such as “sushi bar” with active geolocalisation will tendentially lead to a list of sushi bars in the user’s vicinity. What we are concerned with here (and what Pariser and Sunstein are concerned with) is news-generated information or potentially polarising issues; and we did not find research corroborating the idea that geolocalisation, demographics or previous searches affects the visualization of that kind of information on Google.

13 These studies will be reconsidered more closely and re-evaluated in section 4, when we will reformulate the notion of filter bubble.

14 While our complete re-elaboration of the notion of filter bubble will be done in section 4.4, we want to already clarify here that our understanding of filter bubble as a sociotechnical seclusion is related but distinct from other cognitive mechanisms such as motivated reasoning or motivation biases. Rather, we intend to re-elaborate the notion of filter bubble as an epistemological and affective state occurring while interacting with digital technologies that can in turn trigger psychological responses, biased and motivated rationality processes.

15 With the terms “limits” and “constraints” we are not targeting the faults of some agents. Rather, we are specifically talking about limits - as constraints - that derive from the inevitable situatedness of our potential knowledge and general existence. We simply cannot have a “God’s eye view” on whatever issue we are dealing with because our brains have limited space and our bodies are inevitably situated within specific sociomaterial settings. That being said, of course some people might be more reflexive, curious or open-minded than others, but those epistemic virtues are limited by our natural boundaries as situated organisms. The bubble theses aim to describe some of these unavoidable constraints, and not some defects agents are culpable for.

16 With the term “cognitive irritation” we mean a psychological (and specifically affective) state that is intrinsically and specifically related to the manner agents manage information and form beliefs. This term is already used by Hookway (1998), Pierce (2011) and by Woods (2005) with this meaning.

17 The most straightforward (and sufficient for the purpose of this paper) way to understand this relation would be the following: every Moral Bubble is also an Epistemic Bubble, but the reverse does not stand.

18 The concept of the Ignorance Bubble is notably in tune with various currents that now compose the socalled Ignorance Studies (Gross and McGoey 2015; Peels and Blaauw 2016; Proctor and Schiebinger 2008; Sullivan and Tuana 2007) in which sociological, psychological and philosophical reflections on the concept of ignorance now have found a venue to discuss its complex and interesting features.

19 It is also worth pointing out that, because the ignorance bubble encompasses a structural aspect of human cognition and knowledge-formation, and while the Dunning-Krueger effect applies only to specific circumstances, evidence that seem to falsify the original findings by Dunning and Krueger (Ackerman et al. 2002; Nuhfer et al. 2017) does not undermine the ignorance bubble thesis.

20 With this example we are not in any way implying that this kind of bias or prejudice exists in all religious communities. We do not intend to insult or offend any member of these communities. Rather, this is a mere example, whose purpose is to provide a relatively familiar (to the average reader, at least) instance of a situation where epistemic discomfort is both recognisable and a key factor in the formation of beliefs and their eventual revision. We thank an anonymous peer-reviewer for highlighting the need for this key clarification.

21 We do not think it relevant to discuss whether the (black-boxed) algorithms that structure and provide information in online platforms can be considered intelligent agents. Our view is compatible with the idea that algorithms simply work as an infrastructure that enables users’ information seeking and processing behavior. They do, however, provide the information users consume and interact with: this action may or may not be considered intelligent.

22 It is noteworthy that, in line with the moral bubble thesis, the agent’s cognitive rigidity in front of epistemic discomfort is also motivated by the fact that the belief in question may identify her as a member of her (moral and epistemic) community. Epistemic and (especially) moral embubblement can be shared by a community. For an agent to acknowledge and explicate doubt in this situation would entail that she does not affiliate with her group and its shared values, which constitutes further reasons for her sticking to her own epistemic and moral bubble (see Kahan et al. 2012).

23 This reinforcement of epistemic, ignorance and moral bubble can also be understood as rational (all things considered) if we interpret it as a sort of adaptive preference mechanism. This notion is used to describe the formation of an opinion in front of external pressure or of a reduced number of options (Elster 1983).

24 See also Begby (2020) for a thorough analysis of this confirmation mechanism through confrontation with contrary perspectives.

25 We want to make clear that the bubble theses and our re-conceptualization of the filter bubble do not hinge on any form of determinism (psychological, sociological, metaphysical etc.): while they do describe some epistemological limits of human agency, such limits do not force us to believe certain things instead of others, or make our belief-formation process as such entirely predictable—i.e. these limits are not binding in the same way some *laws* would be. We thank an anonymous peer reviewer for making explicit to us the relevance of this clarification.
